# Supplementary material for: Intermolecular epistasis shaped the function and evolution of an ancient transcription factor and its DNA binding sites
Source: eLife. 2015 Jun 15;4:e07864. doi: 10.7554/eLife.07864 (PMC4500092; doi:10.7554/eLife.07864)
Supplement: Supplementary file 2. — abc/WYK- encoding of sequence characters for linear modeling of genetic effects. (A) One-dimensional vectors for ancestral versus derived state at variable amino acid positions 25, 26, and 29 in the protein are shown. (B) Three-dimensional vectors for A, C, G, or T at variable positions 3 and 4 in the RE are shown. The encoding methods shown in panels A and B ensure that the origin in each vector space will be associated with the mean value of the independent variable (in this case, the delta-G of dissociation) across all the data. (C) Terms used in the linear model using abc/WYK coding. Each row shows the expression for the effect on the independent variable of a nucleotide state, amino acid replacement, or interaction among them. Each genetic effect is calculated using the expression shown and the optimized values of the linear coefficients as described in ‘Materials and methods’. DOI: http://dx.doi.org/10.7554/eLife.07864.012 [file elife07864s002.pdf]

Supplemental Data File 2.

A

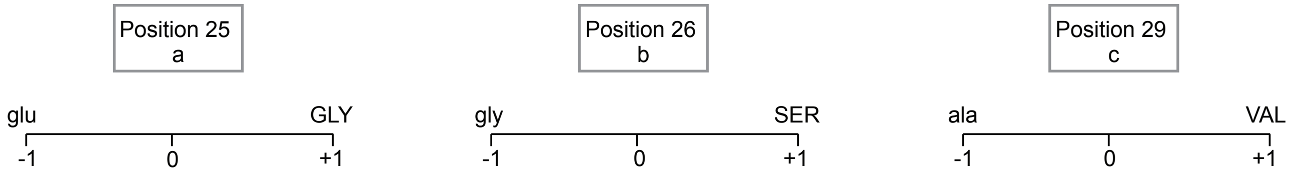

B

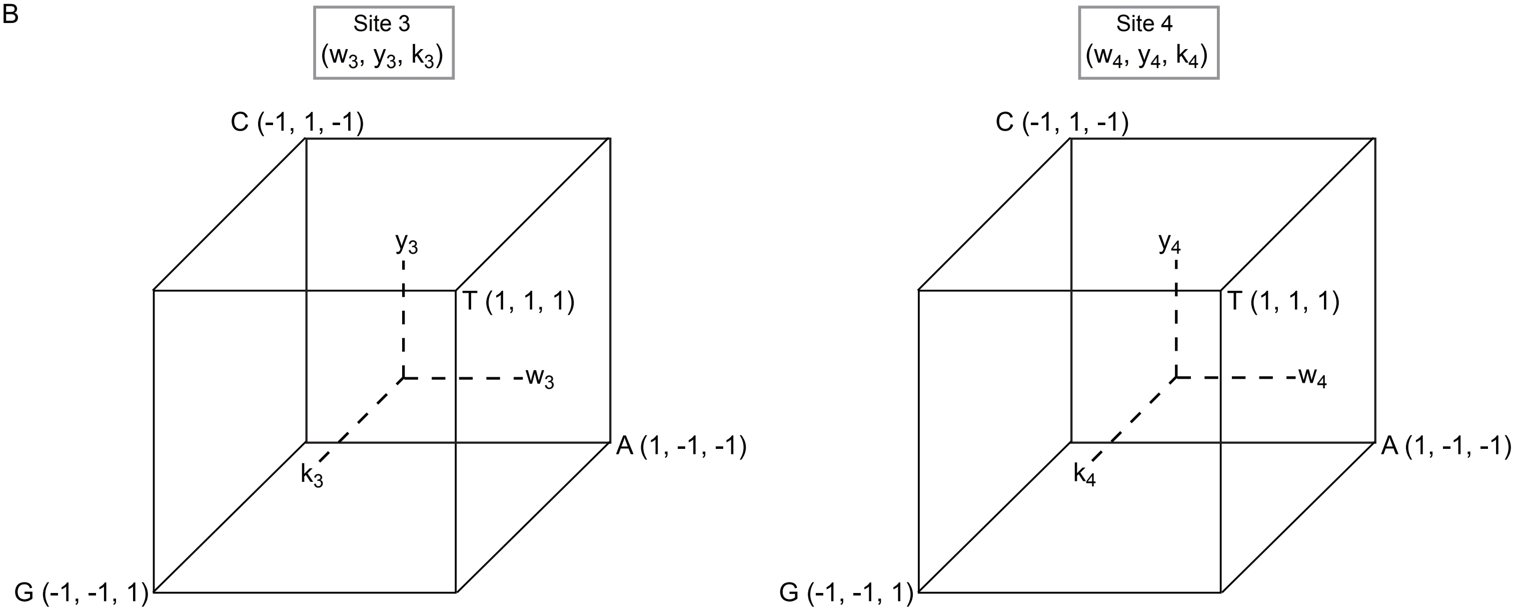

C.

Genetic Term	abc-/WYK-encoded equation
glu25GLY	$2a$
gly26SER	$2b$
ala29VAL	$2c$
A3	$w_3-y_3-k_3$
C3	$-w_3+y_3-k_3$
G3	$-w_3-y_3+k_3$
T3	$w_3+y_3+k_3$
A4	$w_4-y_4-k_4$
C4	$-w_4+y_4-k_4$
G4	$-w_4-y_4+k_4$
T4	$w_4+y_4+k_4$
glu25GLY x gly26SER	$2(ab)$
glu25GLY x ala29VAL	$2(ac)$
gly26SER x ala29VAL	$2(bc)$
A3 x A4	$w_3w_4-w_3y_4-w_3k_4-y_3w_4+y_3y_4+y_3k_4-k_3w_4+k_3y_4+k_3k_4$
A3 x C4	$-w_3w_4+w_3y_4-w_3k_4+y_3w_4-y_3y_4+y_3k_4+k_3w_4-k_3y_4+k_3k_4$
A3 x G4	$-w_3w_4-w_3y_4+w_3k_4+y_3w_4+y_3y_4-y_3k_4+k_3w_4+k_3y_4-k_3k_4$
A3 x T4	$w_3w_4+w_3y_4+w_3k_4-y_3w_4-y_3y_4-y_3k_4-k_3w_4-k_3y_4-k_3k_4$
C3 x A4	$-w_3w_4+w_3y_4+w_3k_4+y_3w_4-y_3y_4-y_3k_4-k_3w_4+k_3y_4+k_3k_4$
C3 x C4	$w_3w_4-w_3y_4+w_3k_4-y_3w_4+y_3y_4-y_3k_4+k_3w_4-k_3y_4+k_3k_4$
C3 x G4	$w_3w_4+w_3y_4-w_3k_4-y_3w_4-y_3y_4+y_3k_4+k_3w_4+k_3y_4-k_3k_4$
C3 x T4	$-w_3w_4-w_3y_4-w_3k_4+y_3w_4+y_3y_4+y_3k_4-k_3w_4-k_3y_4-k_3k_4$
G3 x A4	$-w_3w_4+w_3y_4+w_3k_4-y_3w_4+y_3y_4+y_3k_4+k_3w_4-k_3y_4-k_3k_4$
G3 x C4	$w_3w_4-w_3y_4+w_3k_4+y_3w_4-y_3y_4+y_3k_4-k_3w_4+k_3y_4-k_3k_4$
G3 x T4	$-w_3w_4-w_3y_4-w_3k_4-y_3w_4-y_3y_4-y_3k_4+k_3w_4+k_3y_4+k_3k_4$
T3 x A4	$w_3w_4-w_3y_4-w_3k_4+y_3w_4-y_3y_4-y_3k_4+k_3w_4-k_3y_4-k_3k_4$
T3 x C4	$-w_3w_4+w_3y_4-w_3k_4-y_3w_4+y_3y_4-y_3k_4-k_3w_4+k_3y_4-k_3k_4$
T3 x G4	$-w_3w_4-w_3y_4+w_3k_4-y_3w_4-y_3y_4+y_3k_4-k_3w_4-k_3y_4+k_3k_4$
T3 x T4	$w_3w_4+w_3y_4+w_3k_4+y_3w_4+y_3y_4+y_3k_4+k_3w_4+k_3y_4+k_3k_4$
glu25GLY x A3	$2(aw_3-ay_3-ak_3)$
glu25GLY x C3	$2(-aw_3+ay_3-ak_3)$
glu25GLY x G3	$2(-aw_3-ay_3+ak_3)$
glu25GLY x T3	$2(aw_3+ay_3+ak_3)$
glu25GLY x A4	$2(aw_4-ay_4-ak_4)$
glu25GLY x C4	$2(-aw_4+ay_4-ak_4)$
glu25GLY x G4	$2(-aw_4-ay_4+ak_4)$
glu25GLY x T4	$2(aw_4+ay_4+ak_4)$
gly26SER x A3	$2(bw_3-by_3-bk_3)$
gly26SER x C3	$2(-bw_3+by_3-bk_3)$
gly26SER x G3	$2(-bw_3-by_3+bk_3)$
gly26SER x T3	$2(bw_3+by_3+bk_3)$
gly26SER x A4	$2(bw_4-by_4-bk_4)$
gly26SER x C4	$2(-bw_4+by_4-bk_4)$
gly26SER x G4	$2(-bw_4-by_4+bk_4)$
gly26SER x T4	$2(bw_4+by_4+bk_4)$
ala29VAL x A3	$2(cw_3-cy_3-ck_3)$
ala29VAL x C3	$2(-cw_3+cy_3-ck_3)$
ala29VAL x G3	$2(-cw_3-cy_3+ck_3)$
ala29VAL x T3	$2(cw_3+cy_3+ck_3)$
ala29VAL x A4	$2(cw_4-cy_4-ck_4)$
ala29VAL x C4	$2(-cw_4+cy_4-ck_4)$
ala29VAL x G4	$2(-cw_4-cy_4+ck_4)$
ala29VAL x T4	$2(cw_4+cy_4+ck_4)$
glu25GLY x gly26SER x A3	$2(abw_3-aby_3-abk_3)$
glu25GLY x gly26SER x C3	$2(-abw_3+aby_3-abk_3)$
glu25GLY x gly26SER x G3	$2(-abw_3-aby_3+abk_3)$
glu25GLY x gly26SER x T3	$2(bk_3+abw_3+aby_3+abk_3)$

glu25GLY x gly26SER x A4	$2(abw_4-aby_4-abk_4)$
glu25GLY x gly26SER x C4	$2(-abw_4+aby_4-abk_4)$
glu25GLY x gly26SER x G4	$2(-abw_4-aby_4+abk_4)$
glu25GLY x gly26SER x T4	$2(abw_4+aby_4+abk_4)$
glu25GLY x ala29VAL x A3	$2(acw_3-acy_3-ack_3)$
glu25GLY x ala29VAL x C3	$2(-acw_3+acy_3-ack_3)$
glu25GLY x ala29VAL x G3	$2(-acw_3-acy_3+ack_3)$
glu25GLY x ala29VAL x T3	$2(acw_3+acy_3+ack_3)$
glu25GLY x ala29VAL x A4	$2(acw_4-acy_4-ack_4)$
glu25GLY x ala29VAL x C4	$2(-acw_4+acy_4-ack_4)$
glu25GLY x ala29VAL x G4	$2(-acw_4-acy_4+ack_4)$
glu25GLY x ala29VAL x T4	$2(acw_4+acy_4+ack_4)$
gly26SER x ala29VAL x A3	$2(bcw_3-bcy_3-bck_3)$
gly26SER x ala29VAL x C3	$2(-bcw_3+bcy_3-bck_3)$
gly26SER x ala29VAL x G3	$2(-bcw_3-bcy_3+bck_3)$
gly26SER x ala29VAL x T3	$2(bcw_3+bcy_3+bck_3)$
gly26SER x ala29VAL x A4	$2(bcw_4-bcy_4-bck_4)$
gly26SER x ala29VAL x C4	$2(-bcw_4+bcy_4-bck_4)$
gly26SER x ala29VAL x G4	$2(-bcw_4-bcy_4+bck_4)$
gly26SER x ala29VAL x T4	$2(bcw_4+bcy_4+bck_4)$
glu25GLY x A3 x A4	$2(aw_3w_4-aw_3y_4-aw_3k_4-ay_3w_4+ay_3y_4+ay_3k_4-ak_3w_4+ak_3y_4+ak_3k_4)$
glu25GLY x A3 x C4	$2(-aw_3w_4+aw_3y_4-aw_3k_4+ay_3w_4-ay_3y_4+ay_3k_4+ak_3w_4-ak_3y_4+ak_3k_4)$
glu25GLY x A3 x G4	$2(-aw_3w_4-aw_3y_4+aw_3k_4+ay_3w_4+ay_3y_4-ay_3k_4+ak_3w_4+ak_3y_4-ak_3k_4)$
glu25GLY x A3 x T4	$2(aw_3w_4+aw_3y_4+aw_3k_4-ay_3w_4-ay_3y_4-ay_3k_4-ak_3w_4-ak3y_4-ak_3k_4)$
glu25GLY x C3 x A4	$2(-aw_3w_4+aw_3y_4+aw_3k_4+ay_3w_4-ay_3y_4-ay_3k_4-ak_3w_4+ak_3y_4+ak_3k_4)$
glu25GLY x C3 x C4	$2(aw_3w_4-aw_3y_4+aw_3k_4-ay_3w_4+ay_3y_4-ay_3k_4+ak_3w_4-ak_3y_4+ak_3k_4)$
glu25GLY x C3 x G4	$2(aw_3w_4+aw_3y_4-aw_3k_4-ay_3w_4-ay_3y_4+ay_3k_4+ak_3w_4+ak_3y_4-ak_3k_4)$
glu25GLY x C3 x T4	$2(-aw_3w_4-aw_3y_4-aw_3k_4+ay_3w_4+ay_3y_4+ay_3k_4-ak_3w_4-ak_3y_4-ak_3k_4)$
glu25GLY x G3 x A4	$2(-aw_3w_4+aw_3y_4+aw_3k_4-ay_3w_4+ay_3y_4+ay_3k_4+ak_3w_4-ak_3y_4-ak_3k_4)$
glu25GLY x G3 x C4	$2(aw_3w_4-aw_3y_4+aw_3k_4+ay_3w_4-ay_3y_4+ay_3k_4-ak_3w_4+ak_3y_4-ak_3k_4)$
glu25GLY x G3 x G4	$2(aw_3w_4+aw_3y_4-aw_3k_4+ay_3w_4+ay_3y_4-ay_3k_4-ak_3w_4-ak_3y_4+ak_3k_4)$
glu25GLY x G3 x T4	$2(-aw_3w_4-aw_3y_4-aw_3k_4-ay_3w_4-ay_3y_4-ay_3k_4+ak_3w_4+ak_3y_4+ak_3k_4)$
glu25GLY x T3 x A4	$2(aw_3w_4-aw_3y_4-aw_3k_4+ay_3w_4-ay_3y_4-ay_3k_4+ak_3w_4-ak_3y_4-ak_3k_4)$
glu25GLY x T3 x C4	$2(-aw_3w_4+aw_3y_4-aw_3k_4-ay_3w_4+ay_3y_4-ay_3k_4-ak_3w_4+ak_3y_4-ak_3k_4)$
glu25GLY x T3 x G4	$2(-aw_3w_4-aw_3y_4+aw_3k_4-ay_3w_4-ay_3y_4+ay_3k_4-ak_3w_4-ak_3y_4+ak_3k_4)$
glu25GLY x T3 x T4	$2(aw_3w_4+aw_3y_4+aw_3k_4+ay_3w_4+ay_3y_4+ay_3k_4+ak_3w_4+ak_3y_4+ak_3k_4)$
gly26SER x A3 x A4	$2(bw_3w_4-bw_3y_4-bw_3k_4-by_3w_4+by_3y_4+by_3k_4-bk_3w_4+bk_3y_4+bk_3k_4)$
gly26SER x A3 x C4	$2(-bw_3w_4+bw_3y_4-bw_3k_4+by_3w_4-by_3y_4+by_3k_4+bk_3w_4-bk_3y_4+bk_3k_4)$
gly26SER x A3 x G4	$2(-bw_3w_4-bw_3y_4+bw_3k_4+by_3w_4+by_3y_4-by_3k_4+bk_3w_4+bk_3y_4-bk_3k_4)$
gly26SER x A3 x T4	$2(bw_3w_4+bw_3y_4+bw_3k_4-by_3w_4-by_3y_4-by_3k_4-bk_3w_4-bk_3y_4-bk_3k_4)$
gly26SER x C3 x A4	$2(-bw_3w_4+bw_3y_4+bw_3k_4+by_3w_4-by_3y_4-by_3k_4-bk_3w_4+bk_3y_4+bk_3k_4)$
gly26SER x C3 x C4	$2(bw_3w_4-bw_3y_4+bw_3k_4-by_3w_4+by_3y_4-by_3k_4+bk_3w_4-bk_3y_4+bk_3k_4)$
gly26SER x C3 x G4	$2(bw_3w_4+bw_3y_4-bw_3k_4-by_3w_4-by_3y_4+by_3k_4+bk_3w_4+bk_3y_4-bk_3k_4)$
gly26SER x C3 x T4	$2(-bw_3w_4-bw_3y_4-bw_3k_4+by_3w_4+by_3y_4+by_3k_4-bk_3w_4-bk_3y_4-bk_3k_4)$
gly26SER x G3 x A4	$2(-bw_3w_4+bw_3y_4+bw_3k_4-by_3w_4+by_3y_4+by_3k_4+bk_3w_4-bk_3y_4-bk_3k_4)$
gly26SER x G3 x C4	$2(bw_3w_4-bw_3y_4+bw_3k_4+by_3w_4-by_3y_4+by_3k_4-bk_3w_4+bk_3y_4-bk_3k_4)$
gly26SER x G3 x G4	$2(bw_3w_4+bw_3y_4-bw_3k_4+by_3w_4+by_3y_4-by_3k_4-bk_3w_4-bk_3y_4+bk_3k_4)$
gly26SER x G3 x T4	$2(-bw_3w_4-bw_3y_4-bw_3k_4-by_3w_4-by_3y_4-by_3k_4+bk_3w_4+bk_3y_4+bk_3k_4)$
gly26SER x T3 x A4	$2(bw_3w_4-bw_3y_4-bw_3k_4+by_3w_4-by_3y_4-by_3k_4+bk_3w_4-bk_3y_4-bk_3k_4)$
gly26SER x T3 x C4	$2(-bw_3w_4+bw_3y_4-bw_3k_4-by_3w_4+by_3y_4-by_3k_4-bk_3w_4+bk_3y_4-bk_3k_4)$
gly26SER x T3 x G4	$2(-bw_3w_4-bw_3y_4+bw_3k_4-by_3w_4-by_3y_4+by_3k_4-bk_3w_4-bk_3y_4+bk_3k_4)$
gly26SER x T3 x T4	$2(bw_3w_4+bw_3y_4+bw_3k_4+by_3w_4+by_3y_4+by_3k_4+bk_3w_4+bk_3y_4+bk_3k_4)$
ala29VAL x A3 x A4	$2(cw_3w_4-cw_3y_4-cw_3k_4-cy_3w_4+cy_3y_4+cy_3k_4-ck_3w_4+ck_3y_4+ck_3k_4)$
ala29VAL x A3 x C4	$2(-cw_3w_4+cw_3y_4-cw_3k_4+cy_3w_4-cy_3y_4+cy_3k_4+ck_3w_4-ck_3y_4+ck_3k_4)$
ala29VAL x A3 x G4	$2(-cw_3w_4-cw_3y_4+cw_3k_4+cy_3w_4+cy_3y_4-cy_3k_4+ck_3w_4+ck_3y_4-ck_3k_4)$
ala29VAL x A3 x T4	$2(cw_3w_4+cw_3y_4+cw_3k_4-cy_3w_4-cy_3y_4-cy_3k_4-ck_3w_4-ck_3y_4-ck_3k_4)$
ala29VAL x C3 x A4	$2(-cw_3w_4+cw_3y_4+cw_3k_4+cy_3w_4-cy_3y_4-cy_3k_4-ck_3w_4+ck_3y_4+ck_3k_4)$
ala29VAL x C3 x C4	$2(cw_3w_4-cw_3y_4+cw_3k_4-cy_3w_4+cy_3y_4-cy_3k_4+ck_3w_4-ck_3y_4+ck_3k_4)$
ala29VAL x C3 x G4	$2(cw_3w_4+cw_3y_4-cw_3k_4-cy_3w_4-cy_3y_4+cy_3k_4+ck_3w_4+ck_3y_4-ck_3k_4)$
ala29VAL x C3 x T4	$2(-cw_3w_4-cw_3y_4-cw_3k_4+cy_3w_4+cy_3y_4+cy_3k_4-ck_3w_4-ck_3y_4-ck_3k_4)$

ala29VAL x G3 x A4	$2(-cw_3w_4+cw_3y_4+cw_3k_4-cy_3w_4+cy_3y_4+cy_3k_4+ck_3w_4-ck_3y_4-ck_3k_4)$
ala29VAL x G3 x C4	$2(cw_3w_4-cw_3y_4+cw_3k_4+cy_3w_4-cy_3y_4+cy_3k_4-ck_3w_4+ck_3y_4-ck_3k_4)$
ala29VAL x G3 x G4	$2(cw_3w_4+cw_3y_4-cw_3k_4+cy_3w_4+cy_3y_4-cy_3k_4-ck_3w_4-ck_3y_4+ck_3k_4)$
ala29VAL x G3 x T4	$2(-cw_3w_4-cw_3y_4-cw_3k_4-cy_3w_4-cy_3y_4-cy_3k_4+ck_3w_4+ck_3y_4+ck_3k_4)$
ala29VAL x T3 x A4	$2(cw_3w_4-cw_3y_4-cw_3k_4+cy_3w_4-cy_3y_4-cy_3k_4+ck_3w_4-ck_3y_4-ck_3k_4)$
ala29VAL x T3 x C4	$2(-cw_3w_4+cw_3y_4-cw_3k_4-cy_3w_4+cy_3y_4-cy_3k_4-ck_3w_4+ck_3y_4-ck_3k_4)$
ala29VAL x T3 x G4	$2(-cw_3w_4-cw_3y_4+cw_3k_4-cy_3w_4-cy_3y_4+cy_3k_4-ck_3w_4-ck_3y_4+ck_3k_4)$
ala29VAL x T3 x T4	$2(cw_3w_4+cw_3y_4+cw_3k_4+cy_3w_4+cy_3y_4+cy_3k_4+ck_3w_4+ck_3y_4+ck_3k_4)$
